# Human Milk Blocks DC-SIGN–Pathogen Interaction via MUC1

**DOI:** 10.3389/fimmu.2015.00112

**Published:** 2015-03-13

**Authors:** Nathalie Koning, Sabine F. M. Kessen, J. Patrick Van Der Voorn, Ben J. Appelmelk, Prescilla V. Jeurink, Leon M. J. Knippels, Johan Garssen, Yvette Van Kooyk

**Affiliations:** ^1^Department of Molecular Cell Biology and Immunology, VU University Medical Center, Amsterdam, Netherlands; ^2^Department of Pathology, VU University Medical Center, Amsterdam, Netherlands; ^3^Department of Medical Microbiology and Infection Control, VU University Medical Center, Amsterdam, Netherlands; ^4^Immunology, Danone Research – Centre for Specialised Nutrition, Wageningen, Netherlands; ^5^Faculty of Science, Utrecht Institute for Pharmaceutical Sciences, University Utrecht, Utrecht, Netherlands

**Keywords:** DC-SIGN, human milk, immune modulation, intestine, mucin

## Abstract

Beneficial effects of breastfeeding are well-recognized and include both immediate neonatal protection against pathogens and long-term protection against allergies and autoimmune diseases. Although several proteins have been identified to have anti-viral or anti-bacterial effects like secretory IgA or lactoferrin, the mechanisms of immune modulation are not fully understood. Recent studies identified important beneficial effects of glycans in human milk, such as those expressed in oligosaccharides or on glycoproteins. Glycans are recognized by the carbohydrate receptors C-type lectins on dendritic cell (DC) and specific tissue macrophages, which exert important functions in immune modulation and immune homeostasis. A well-characterized C-type lectin is dendritic cell-specific intercellular adhesion molecule-3-grabbing non-integrin (DC-SIGN), which binds terminal fucose. The present study shows that in human milk, MUC1 is the major milk glycoprotein that binds to the lectin domain of DC-SIGN and prevents pathogen interaction through the presence of Lewis x-type oligosaccharides. Surprisingly, this was specific for human milk, as formula, bovine or camel milk did not show any presence of proteins that interacted with DC-SIGN. The expression of DC-SIGN is found in young infants along the entire gastrointestinal tract. Our data thus suggest the importance of human milk glycoproteins for blocking pathogen interaction to DC in young children. Moreover, a potential benefit of human milk later in life in shaping the infants immune system through DC-SIGN cannot be ruled out.

## Introduction

Inflammatory diseases like allergies and autoimmune diseases are increasing in the Western world. These disorders are associated with a disturbed immune balance, feeding the necessity to unravel mechanisms of immune tolerance induction, specifically early in life. For years, it is known that breastfeeding has beneficial effects for infants at young age, for example, by strongly reducing the mortality rate from infections by common pathogens. Moreover, breastfed individuals showed, although with controversies, a lower risk of developing allergies and autoimmune disorders such as type 1 diabetes, Crohn’s disease, and juvenile rheumatoid arthritis later in life (1–4). For a long time, protection against pathogens was attributed to secretory IgA, which could capture pathogens from the gastrointestinal system of the child. Later, also milk components were identified as possible immune modulators. For example, the major milk protein lactoferrin is well known for its anti-viral and anti-bactericidal activity (5), and similar effects have been described for lysozyme (6). Other immunologically interesting components of human milk include growth hormones, cytokines, and glycans.

Glycans in human milk are abundantly present in oligosaccharides or on glycoproteins. These glycans may play an important role in protective immunity in young children. It has been demonstrated that the incidence of diarrhea caused by different pathogens is inversely related to the type and amount of fucose-type glycans in human milk that the infants received (7, 8). Interestingly, high concentrations of such glycans were also significantly associated with a lower incidence of respiratory disease in children (9), suggesting beneficial systemic effects as well.

Glycans are recognized by C-type lectins expressed mostly by myeloid antigen presenting cells such as macrophages and dendritic cells (DCs). C-type lectins are carbohydrate receptors that bind a variety of glycan moieties in a calcium-dependent manner. They belong to the family of antigen uptake receptors, as they recognize cell surface glycans on many viruses, bacteria, and parasites, but may bind and respond to glycans on self-proteins as well. A well-characterized C-type lectin on DCs is dendritic cell-specific intercellular adhesion molecule-3-grabbing non-integrin (DC-SIGN). Ligands for DC-SIGN include fucosylated structures, like the Lewis antigens, as well as mannosylated moieties. As antigen uptake receptor, DC-SIGN shuttles antigen in the intracellular compartments of DCs to load antigen on MHC class I and II molecules to stimulate antigen-specific CD8 and CD4 T cell responses, respectively (10–13). On the other hand, DC-SIGN recognizes a wide variety of pathogens that is not necessarily presented to T cells but rather modulates immune responses. For example, DC-SIGN signaling following binding to *Mycobacterium tuberculosis*, *Borrelia burgdorferi*, or HIV results in immune inhibition through Raf-1 activation, prolonged Nf-κB activation, high IL-10 production, or decreased expression of proinflammatory cytokines (14–16). The diversity of DC-SIGN actions was further shown by its ability to promote either Th1 or Th2 responses, dependent on the phase variant of *Helicobacter pylori* (17). Also, probiotics have been reported to interact with DC-SIGN, which instead resulted in the induction of regulatory T cell responses (18). In the case of a pathogen interacting with DC-SIGN, the simultaneously triggering of specific toll-like receptors (TLR), and the interplay between DC-SIGN and TLR signaling is important in the differential outcome of the immune response. DC-SIGN is thus an innate signaling receptor that dependent on the type of glycan it interacts with (mannose or fucose) interferes with TLR signaling (19). Based on its specificity for self-glycosylated proteins, such as CEA, MUC1, MUC6, butyrophilin, DC-SIGN has been considered important for maintenance of immune homeostasis (20).

The contributions of the gastrointestinal tract in shaping immunity are undeniable. Mucus layers, anti-bacterial proteins, and numerous amounts of microbial communities, termed the microbiota, all cooperate in protecting the host while providing metabolic benefits, as excellently reviewed by Hooper et al. (21). Importantly, DCs are localized in all areas of the gastrointestinal tract. Equipped with C-type lectins and other molecules for recognizing, internalizing, and presenting antigens, DCs are the primary cells to initiate various immune responses, including anergy. Despite all indications that abundant glycans in human milk provide important benefits to the infant, underlying biological mechanisms have not yet been addressed. Therefore, we aimed to study the interaction of human milk with C-type lectins on DCs. In adults, DC-SIGN is expressed on a subpopulation of DCs in the intestinal mucosa (22, 23). However, information on DC-SIGN expression in the gastrointestinal tract of neonates is scarce. Data from the present study show that human milk strongly interacts with DCs through DC-SIGN expressed in the entire gastrointestinal tract of young infants. Furthermore, our data suggest that this interaction is dependent on Lewis x present on the glycoprotein mucin 1 (MUC1). We demonstrate this to be a potent mechanism in blocking pathogen interaction with DCs and suggest this to be an important mechanism of the capability of human milk to modulate the infant’s immunity, presumably with long-term health benefits.

## Materials and Methods

### Milk samples

Human milk from 40 mothers was provided by the European Milk Bank Association (EMBA, Milan, Italy). Human milk samples, as well as bovine milk (Campina, The Netherlands), formula milk (Nutricia Nutrilon 1, Danone, reconstituted according to the manufacturers instructions), and camel milk (camel farm Smits, Berlicum, The Netherlands) were skimmed by obtaining the aqueous phase after three consecutive rounds of centrifugation at 680 × *g* for 10 min at 4°C. The samples were stored at −80°C. For use in cell culture experiments, skimmed milk samples were filter-sterilized. To fractionate human milk, the aqueous layer (50 ml) was freeze-dried overnight and dissolved in 15 ml water, mixed with 15 ml N-butanol and 30 ml di-isopropyl ether and incubated at 4°C for 2 h, rolling. After centrifugation, the upper (organic) layer was removed and aqueous layer mixed again with 30 ml di-isopropyl ether for 2 h incubation at 4°C. After centrifugation, the aqueous layer was collected and freeze-dried overnight. Twenty milliliters of PBS were added and proteins were solubilized for 1 h by sonication and filtered through a 0.45 μm filter. Finally, milk proteins were separated on a gel filtration column [Sepharose 6 (10 × 300), GE Healthcare Europe].

### Reagents

Recombinant DC-SIGN and MGL proteins consisted of the extracellular region (containing the carbohydrate recognition domain) fused with an immunoglobulin Fc tail for detection in ELISA and were produced by 293 T cells as described previously (24). The DC-SIGN blocking antibody AZN-D1 and 1G6.6 antibody for MGL were purified from hybridoma supernatant using a protein A sepharose FF column (Amersham). Lactoferrin isolated from human milk was obtained from Sigma-Aldrich. MUC1 was detected by clone 214D4 (provided by John Hilkens, Netherlands Cancer Institute, Amsterdam) specifically recognizing the amino acid sequence PDTR in the extracellular domain of MUC1 (glycosylation independent) and was biotinylated using a biotinylation kit (Pierce). MUC4 was detected by clone 8G7, κ-casein by clone A-14 (Santa Cruz Biotechnology, Inc.), α-lactalbumin by clone F20.16 (Novus Biologicals), Lewis x by clone P12 and Lewis y by clone F3 (Calbiochem), CD83-PE by clone L307.4, CD86-PE by clone 2331, HLA-DR-PE by clone L243 (BD Pharmingen), and DC-SIGN by clone DC-28 (gift of R. Doms, University of Pennsylvania, Philadelphia, PA, USA).

### ELISA

Maxisorp ELISA plates (NUNC) were either coated directly with skimmed milk or fractionated milk samples (in 1:10 dilution, unless stated otherwise) in 0.2 M NaHCO_3_ buffer overnight at 4°C, or in case of human milk protein capturing ELISA, with purified lactoferrin (100 ng/ml) or with antibodies to MUC1 (unbiotinylated, 3 μg/ml), MUC4 (40 μg/ml), κ-casein (20 μg/ml), or α-lactalbumin (30 μg/ml). The plates were blocked in Tris-sodium buffer containing 1% BSA, followed by incubation with skimmed human milk in case of milk protein capture. For C-type lectin binding, plates were washed in Tris-sodium-0.05%Tween and incubated with DC-SIGN-Fc or MGL-Fc for 1 h at room temperature (RT). For MUC1 detection, plates were incubated with biotinylated MUC1 antibody (2 μg/ml). To detect Lewis-antigen expression following MUC1 capture, plates were incubated with antibodies to Lewis x or Lewis y (0.5 μg/ml). After washing, binding of C-type lectins was detected by PO-labeled goat anti-human IgG, binding of MUC1 by PO-labeled streptavidin and binding of Lewis antigens by PO-labeled goat anti-mouse IgM (Jackson Immunoresearch). The reaction was visualized in 100 μg/ml 3,3′-5,5′-tetramethylbenzidine (TMB) substrate (Sigma-Aldrich) and optical density was measured by a microplate absorbance spectrophotometer (Biorad) at 450 nm.

### Cells

Human immature DCs were generated from monocytes isolated from buffy coats of healthy donors (Sanquin, Amsterdam, The Netherlands) by culturing the cells for 4–5 days in RPMI 1640 medium (Invitrogen) containing 10% fetal calf serum and in the presence of IL-4 and GM-CSF (500 and 800 U/ml, respectively, Biosource). Monocyte-derived DCs were matured in the presence of LPS (10 ng/ml, Sigma-Aldrich) for 24 h and co-incubated with sterilized human milk (1:10 dilution) and AZN-D1 (20 μg/ml). Next, cells were either washed and incubated with indicated antibodies for detection in flow cytometry and analyzed using FlowJo Software, or were used for quantitative PCR.

### Cell adhesion assay

Nunc-Immuno Maxisorp plates were coated with milk (1:10 dilution unless stated otherwise) in NaHCO_3_ buffer (0.2 M) o/n at 4°C. After washing and blocking the NUNC plate in TSM/BSA (1%), DCs labeled with Calcein-AM (Molecular Probes) were added in the presence or absence of the calcium chelator EGTA (10 mM), AZN-D1 (20 μg/ml), or 1G6.6 (20 μg/ml) and incubated for 90 min at 37°C. The non-adherent cells were gently washed away and the adherent cells were lysed in 50 mM Tris-HCL/0.1%SDS. Fluorescence was quantified on a Fluostar spectrofluorimeter (BMG Labtech) at 485/520 nm.

### Bacteria blocking assay

Dendritic cells (1 × 10^5^ cells/well) were incubated with filter-sterilized milk (1:10 dilution), AZN-D1 (10 μg/ml), 1g6.6 (10 μg/ml), or Tris-sodium buffer alone for 45 min at 37°C. FITC-labeled DC-SIGN binding variant of *Neisseria gonorrhoeae* (25) or FITC-labeled *H. pylori* (phase variants J223.3 and J223.8) (17) were co-incubated with the DCs in a 25:1 (bacteria:DC) ratio for another 45 min at 4°C. Cells were washed in Tris-sodium buffer and FITC-positive DCs were quantified using a flow cytometer (Facscan, BD) and FlowJo Software (Tree Star, Inc.).

### Quantitative PCR

Cells were lysed for RNA isolation (mRNA capture kit, Roche) and cDNA synthesis (Reverse Transcription System, Promega), according to manufacturers instructions, after which the cDNA was stored in −20°C until further use. PCR reactions were performed with the SYBR Green method in an ABI 7900HT sequence detection system (Applied Biosystems, USA). Primer sets to the following human target genes were used: IL-10 (fw: 5′-gaggctacggc gctgtcat-3′, rv: 5′-ccacggccttgctcttgtt-3′), TNF (fw: 5′-tctcgaaccc cgagtgaca-3′, rv: 5′-tgaggtacaggccctctgatg-3′), IL-6 (fw: 5′-tgcaataa ccacccctgacc-3′, rv: 5′-tgcgcagaatgagatgagttg-3′), IL-12p35 (fw:5′-ccactccagacccaggaatgt-3′, rv: 5′-gcaggttttgggagtggtga-3′), and IL-23p19 (fw: 5′-gcttgcaaaggatccacca-3′, rv: 5′-tccgatcctagcagcttct ca-3′). Expression of the target gene was normalized to GAPDH (fw: 5′-ccatgttcgtcatgggtgtg-3′, rv: 5′-ggtgctaagcagttggtggtg-3′), based on a protocol for the definition of the optimal endogenous reference gene (26).

### Immunohistochemistry

Paraffin embedded sections of stomach, duodenum, ileum, colon, and rectum were derived from biopsies (taken for reasons such as diarrhea, suspicion of celiac disease or Hirschsprung’s disease) or resections (of congenital malformations, such as atresia) from three different infants per anatomical region. The average age was 6.5 months (range: 1 week–17 months). Material was obtained with informed consent and has been approved by the Ethical Committee VU University Medical Center (Amsterdam, The Netherlands) Biobank Unit Pathology BUP2012-24. The absence of tissue abnormalities was confirmed by a clinical pathologist (JPvdV). Sections were deparaffinized and hydrated in a xylene to alcohol to distilled water series, followed by heat-induced epitope retrieval in sodium-citrate buffer (pH 7.6). Endogeneous peroxidase was blocked in blocking buffer (DAKO EnVision). Sections were incubated in 10% normal goat serum before incubating with anti-DC-SIGN antibody (DC-28) for 1 h RT. Next, sections were incubated with HRP-labeled secondary antibody (DAKO EnVision) and staining was visualized with DAB (DAKO EnVision). Sections were counterstained with hematoxylin, dehydrated, and mounted in Entallan. All washing steps between incubations were performed in TSM/0.05%Tween.

### Statistical analysis

Data were analyzed for significance by one-way analysis of variance and Spearman’s correlation test, using GraphPad Prism software (version 5.01). Only *p*-values <0.05 were considered significant.

## Results

### Human milk binds DC-SIGN on DCs

**Figure 1:**
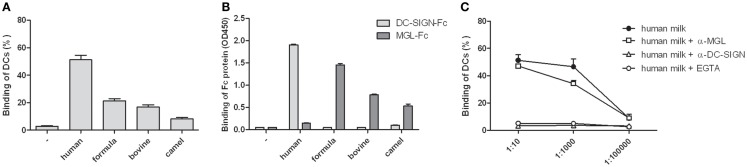
**(A)** Binding of monocyte-derived dendritic cells to skimmed human, formula, bovine, or camel milk. **(B)** Binding of skimmed milk to DC-SIGN-Fc or MGL-Fc. **(C)** Binding of monocyte-derived dendritic cells to skimmed human milk in different dilutions in the presence of EGTA, DC-SIGN blocking antibody AZN-D1, or MGL blocking antibody 1G6.6.Three independent experiments were performed. Data are indicated as mean ± SD.

To establish the presence of DC-SIGN binding glycoproteins in human milk, interaction of human milk with DCs was tested in a cell adhesion assay. DCs strongly attached to coated human milk, and to a lesser extent to coated bovine, camel, or formula milk (Figure 1A). Because glycoproteins are abundantly present in human milk, we hypothesized that binding is mediated by glycan receptors, i.e., C-type lectins. Using Fc constructs, we indeed show that human milk strongly interacted with DC-SIGN-Fc, but not to MGL-Fc. In contrast, bovine, camel, and formula milk bound to MGL-Fc, but not to DC-SIGN-Fc (Figure 1B). No differences in C-type lectin binding were observed between unskimmed, skimmed, and sterilized milk samples (data not shown). A cell adhesion assay confirmed that the strong binding of human milk with DCs was DC-SIGN dependent, because binding was completely blocked by a DC-SIGN blocking antibody, whereas blocking MGL had no effect (Figure 1C). These data show that human milk specifically interacts with DCs via DC-SIGN.

### Lewis x on MUC1 in human milk binds DC-SIGN

**Figure 2:**
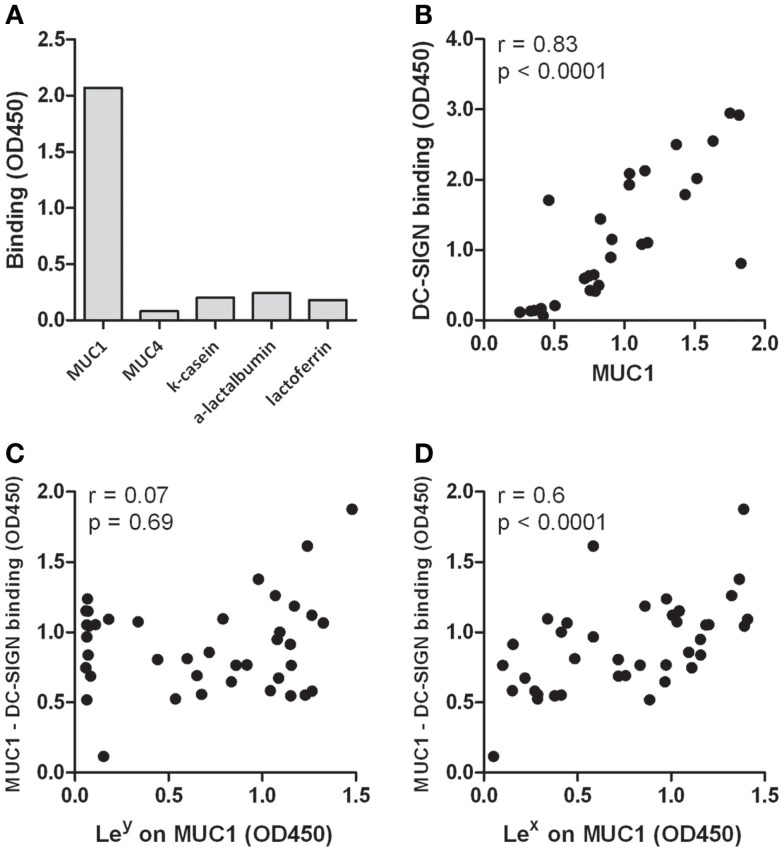
**(A)** Binding of milk proteins to DC-SIGN. MUC1, MUC4, κ-casein, and α-lactalbumin were captured from human milk using antibodies, whereas lactoferrin was used in purified form. One representative experiment out of three is presented. **(B)** Correlation of MUC1 levels with DC-SIGN binding in fractionated human milk. Human milk was fractionated into 30 fractions, based on protein size. MUC1 levels and DC-SIGN binding was tested in ELISA. **(C)** Correlation of Lewis x levels on MUC1 in 40 milk donors with the capacity of captured MUC1 binding to DC-SIGN. **(D)** Correlation of Lewis y levels on MUC1 in 40 milk donors with the capacity of captured MUC1 binding to DC-SIGN. Typical experiment out of two is presented.

Different milk proteins were tested for their ability to bind DC-SIGN. Antibodies to the milk proteins mucin 1 (MUC1), MUC4, α-lactalbumin, and κ-casein were coated on plates to capture the proteins from human milk. Lactoferrin was used as purified protein. MUC1 was the only milk protein showing potent DC-SIGN binding (Figure 2A). Next, human milk was fractionated into 30 fractions, based on protein size, after which we determined the presence of MUC1 as well as the amount of DC-SIGN binding in these fractions. A strong positive correlation between the level of MUC1 and the capacity to bind DC-SIGN (*r* = 0.8, *p* < 0.0001, Figure 2B) suggests that MUC1 is a major human milk component binding to DC-SIGN.

Next, we analyzed well-known ligands for DC-SIGN, Lewis x, and Lewis y, on MUC1 in milk samples derived from 40 milk donors. Both Lewis x and y could be detected on MUC1, but expression levels are donor-dependent. Interestingly, levels of Lewis x on MUC1 positively correlated with binding of MUC1 to DC-SIGN (*r* = 0.6, *p* < 0.0001, Figure 2C), but Lewis y levels on MUC1 did not (*r* = 0.07, *p* = 0.69, Figure 2D). Importantly, no correlation was found between the level of Lewis antigens and the level of MUC1 in these samples (Lewis x: *r* = 0.27, *p* = 0.1; Lewis y: *r* = 0.03, *p* = 0.8), indicating that higher MUC1 levels do not inevitably contain higher Lewis antigen levels and thus reflects individual variability in Lewis antigen expression. These data therefore strongly suggest that human milk binds DC-SIGN via Lewis x on MUC1.

### Interaction of human milk with DC-SIGN does not affect DC immune responses

**Figure 3:**
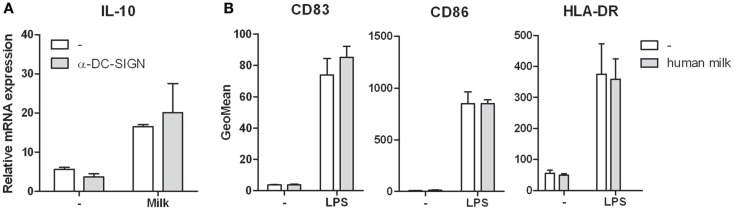
**(A)** IL-10 expression in human monocyte-derived dendritic cells incubated in human milk with and without the blocking DC-SIGN antibody AZN-D1. Expression is normalized to GAPDH. **(B)** Cell surface expression of the maturation markers CD83, CD86, and HLA-DR on human monocyte-derived dendritic cells following incubation with human milk and after LPS stimulation. Data are indicated as mean ± SD of three experiments performed.

DC-SIGN triggering has been described to modulate immune responses (14). We therefore studied the effect of milk-DC-SIGN interaction on DC-mediated responses. Although LPS activated DCs incubated with human milk showed upregulation of IL-10 mRNA and protein levels, this was not attributed to DC-SIGN as blocking DC-SIGN did not inhibit IL-10 induction (Figure 3A). Similar results were obtained for TNF (data not shown). Human milk did not affect the expression of IL-6, IL-12p35, and IL-23p19 (data not shown). Furthermore, human milk did neither induce DC maturation nor affected LPS-mediated DC maturation (Figure 3B) or T cell proliferation (data not shown).

### MUC1 in human milk blocks pathogen interactions with DCs

**Figure 4:**
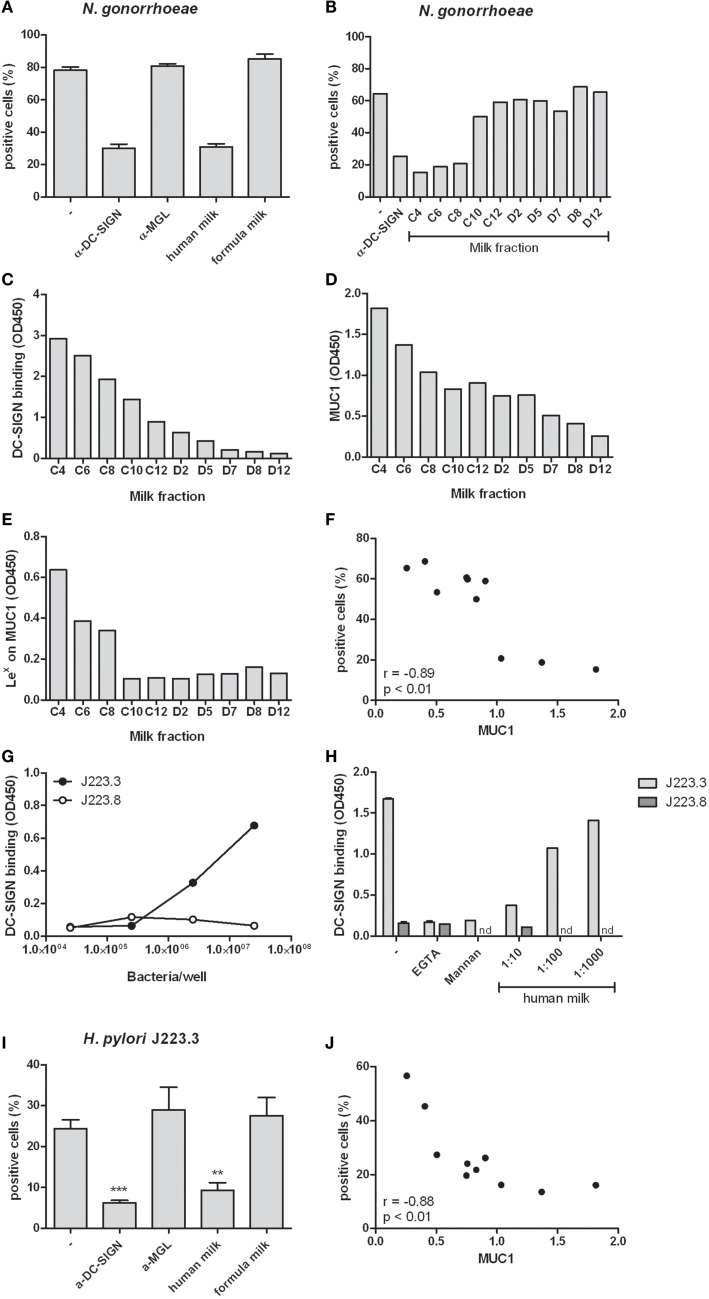
**(A)** Binding of *N. gonorrhoeae* to human monocyte-derived dendritic cells in the presence of the DC-SIGN blocking antibody AZN-D1, MGL blocking antibody 1g6.6, human milk or formula milk. Three independent experiments were performed. **(B)** Binding of *N. gonorrhoeae* to monocyte-derived dendritic cells in the presence of fractionated milk samples. **(C)** DC-SIGN binding capacity of fractionated milk samples in ELISA. **(D)** MUC1 levels in fractionated milk samples detected by ELISA. **(E)** Lewis x expression on MUC1 captured from fractionated milk samples in ELISA. **(F)** Correlation of MUC1 levels in fractionated milk samples and the capacity of these samples to inhibit *N. gonorrhoeae* binding to monocyte-derived dendritic cells. **(G)** Binding of phase variants J223.3 and J223.8 from *H. pylori* to DC-SIGN-Fc in ELISA. **(H)** Binding of *H. pylori* phase variants to DC-SIGN-Fc in the presence of the calcium chelator EGTA, the DC-SIGN ligand mannan and human milk. **(I)** Binding of *H. pylori* phase variant J223.3 to monocyte-derived dendritic cells in the presence of anti- DC-SIGN or anti-MGL antibody, human or formula milk. **(J)** Correlation of MUC1 levels in fractionated milk samples and the capacity of these samples to inhibit *H. pylori* J223.3 binding to monocyte-derived dendritic cells. Data are indicated as mean ± SD of three experiments performed.

We hypothesized that the abundantly present glycoproteins in human milk may have protective properties by preventing pathogen interactions with DCs. To test this hypothesis, we incubated DCs with the DC-SIGN binding variant of *N. gonorrhoeae*. Binding of *N. gonorrhoeae* was inhibited in the presence of a blocking DC-SIGN antibody, but not when MGL was blocked (Figure 4A). Interestingly, human milk inhibited *N. gonorrhoeae* binding to DCs to a similar extent as the DC-SIGN blocking antibody. As expected, formula milk, which does not bind to DC-SIGN but rather binds MGL (Figure 1B), had no effect on bacteria binding. Because human milk binding to DC-SIGN is dependent on Lewis x on MUC1 (Figure 2), we incubated DCs with *N. gonorrhoeae* in the presence of fractionated milk samples containing different amounts of MUC1. Indeed, the fractions that inhibited bacteria binding to DCs (Fraction C4, C6, and C8, Figure 4B), showed strongest DC-SIGN binding in ELISA (Figure 4C), contained highest levels of MUC1 (Figure 4D) as well as the highest amount of Lewis x present on MUC1 (Figure 4E), reflected in a highly significant negative correlation between the percentage of bacteria-positive cells and the levels of MUC1 (Figure 4F). To see whether these observations also apply to a relevant gastrointestinal pathogen, we tested the gastrointestinal pathogen *H. pylori*. We used two phase variants of *H. pylori* that were previously shown to bind differentially to DC-SIGN (17). We confirmed that phase variant J223.3 binds to DC-SIGN but phase variant J223.8 does not (Figure 4G), as a result of the lack of Lewis y and x (17). In a competition assay, we further demonstrate that J223.3 binding to DC-SIGN was inhibited by the negative controls mannan and the calcium chelator EGTA and by human milk in a dose dependent manner (Figure 4H). Like shown for *N. gonorrhoeae*, human milk also blocks *H. pylori* J223.3 binding to DCs to a similar extent as the DC-SIGN blocking antibody, and, in line with Figure 4A, anti-MGL or formula milk did not affect bacteria binding to DCs (Figure 4I). A strong and significant negative correlation between J223.3-positive DCs and MUC1 levels demonstrates that MUC1 potently inhibits bacteria binding to DCs (Figure 4J).

### DC-SIGN expression in intestinal tract of young infants

**Figure 5:**
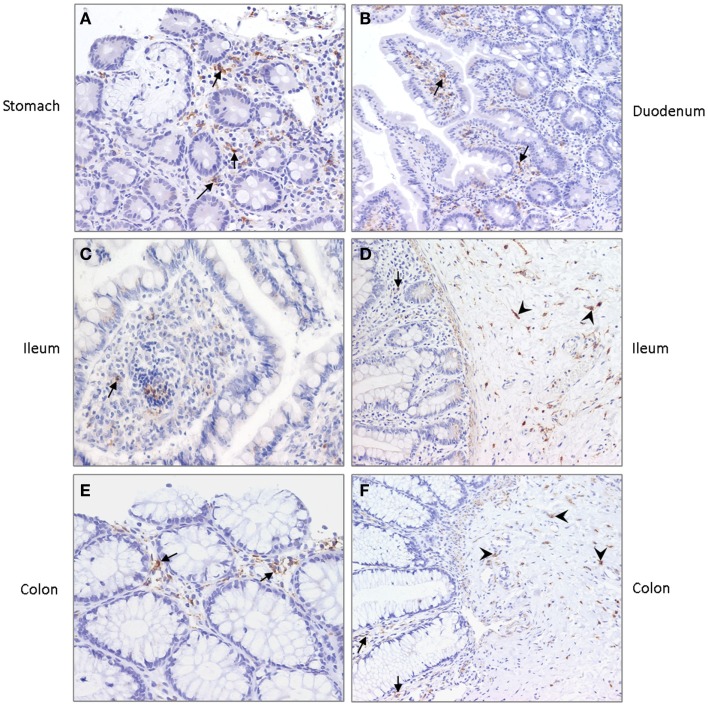
**DC-SIGN expression in (A) stomach, (B) duodenum, (C,D) ileum, and (E,F) colon of young infants**. Arrows indicate DC-SIGN positive cells in the lamina propria and arrowheads point to positive cells in the submucosa. Nuclei are stained in blue. Magnification ×20.

To investigate the expression of DC-SIGN in the gastrointestinal tract of young infants, we set out to stain various tissues obtained from young infant. Irregular shaped DC-SIGN positive cells were detected in the lamina propria of the stomach, duodenum, ileum, and colon (Figure 5). Expression, however, was not uniform, with each anatomical position containing areas with both high and low numbers of DC-SIGN positive cells. In addition, strong DC-SIGN expression was observed on cells in the submucosa of the ileum and colon, like reported previously in adult tissue (22). The DC-SIGN positive cells in the submucosa had an irregular shaped morphology consistent with DCs, and some cells were in close vicinity of blood vessels.

## Discussion

A wide spectrum of biologically active agents present in human milk extensively promotes the development of the neonatal immature intestine and immune system. The beneficial effects include maturation of the gut barrier, favorable colonization by probiotics, development of tolerance, and thereby related protection against allergies and autoimmune disorders later in life and immediate protection against different pathogens early in life. In the present study, we show how the heavily glycosylated protein MUC1 in human milk prevents pathogen interaction via Lewis x interaction with DC-SIGN, which is expressed on DCs throughout the gastrointestinal tract of young infants. Because C-type lectins are vital in immune modulation and in the maintenance of immune homeostasis, these data provide further clues for potential mechanisms for proper maturation and tolerization of the neonatal immune system.

In the adult human gastrointestinal tract, DCs are located in the submucosa and in the lamina propria, where they can form protrusions to sample antigens from the gut lumen (27). Several DC subsets with their own ontogeny have been identified (22, 28), however, data on the presence and type of DCs in the infants’ intestines are very scarce. Here, we identified DC-SIGN expressing cells in the lamina propria of the stomach, duodenum, ileum, colon, and rectum as well as in the submucosa of the ileum, colon, and rectum of young infants. These data are in line with DC-SIGN expression previously found in the lamina propria and associated lymphoid aggregates in fetal tissue (29). DC-SIGN, like most C-type lectins, act as antigen receptors that are able to capture and internalize glycosylated self and foreign antigens from the micro-environment. Although we did not observe a direct DC-SIGN-mediated effect on DC cytokine production or maturation in this study, DC-SIGN is considered important in tolerance induction and maintenance of immune homeostasis by modifying T cell responses (14, 20). Interestingly, mouse studies demonstrate that antigen exposure via maternal milk prevents the development of allergic asthma (30–32). Because of the presence of DC-SIGN in the infant’s intestine and its immunomodulatory capacity, we speculate an important role for DC-SIGN in capturing antigens from human milk, thereby inducing tolerance. In fact, it has been shown that the mouse homolog of DC-SIGN, SIGNR1, conditions the mucosal immune system to reduce the anaphylactic response triggered by food allergens (33). In addition, also different species of probiotic lactobacilli drive the development of regulatory T cells via specific DC-SIGN interaction (18). Conclusively, these data support an important role for DC-SIGN in the proper development of the neonatal immune system.

Whereas binding of antigens and probiotics in the intestine is important for the induction of tolerance, human milk further protects the neonate by preventing interaction with pathogens like *Salmonella*, *Shigella*, *Vibrio cholerae*, *Escherichia coli*, polioviruses, rotavirus, and respiratory syncytial virus (RSV) (34). We here show that MUC1 is a major milk protein preventing the interaction of bacteria with DC-SIGN on DCs, via Lewis x, but not Lewis y moieties. As this effect was observed for two different and unrelated bacteria, it is highly likely that this mechanism applies to other DC-SIGN binding bacteria as well. Importantly, in our earlier work, we have demonstrated that fucosylated milk components, and in particular MUC1 interacted with DC-SIGN and was shown to inhibit DC-mediated transfer of HIV-1 (35–37). In addition to MUC1, also κ-casein inhibits adhesion of *H. pylori* to human gastric mucosa (38), but our data indicate that κ-casein does not interact with DC-SIGN. Indeed, inhibition of *Streptococcus pneumoniae* and *H*. *influenzae* binding to airway epithelium by κ-casein seemed rather to depend on GlcNAc containing saccharides (39). Other milk proteins that via a diversity of mechanisms have demonstrated potent protective effects include bile-salt-stimulated lipase (BSSL) (40), soluble CD14 (41), immunoglobulins (42), lsyozyme (6), and lactoferrin (5). Taken together, these milk proteins may exert their protective effects alone, but it is more likely that they act in synergy, where the current findings suggest MUC1–DC-SIGN interaction as a significant mechanism in this system, via Lewis-type antigens expressed on MUC1.

The importance of glycans in human milk is stressed by studies showing strong inverse correlations between the incidence of diarrhea caused by different pathogens such as *Campylobacter*, Norovirus, and Calicivirus, and the levels and types of human milk oligosaccharides (7, 8). In particular, 2-linked fucoses seemed responsible for the protection. Although these studies focused on oligosaccharides, our data indicate the importance of fucose moieties on glycoproteins as MUC1 as well. Furthermore, we corroborate an earlier extensive glycan analysis on human milk-derived MUC1 revealing the presence of terminal fucoses (43). Interestingly, such fucoses are not expressed in bovine milk (44), confirming our data that bovine milk does not bind DC-SIGN, and may therefore not have similar pathogen inhibitory mechanisms as human milk. The high variability of fucoses in human milk depends on the genetically determined FUT2 expression of the mother. Failure to express this enzyme results in the inability to attach 2-linked fucoses during the glycosylation process. Besides inter-individual variability, secretion of the fucosylated glycans also shows large variations during the course of lactation (40). In addition to the relationship with diarrhea, it would be highly interesting to study whether these individual variations affects the development of the immune system and the immunological outcome later in life.

At present, we have only begun to realize the importance of our gastrointestinal tract in shaping the immune system. Understanding the immunological benefits of human milk compounds is highly valuable in order to stimulate proper development of the infant’s immune system. Additionally, infant formulas could be supplemented with glycosylated compounds based on human milk, aiming to provide the same beneficial effects. Most favorable, specific human milk compounds may be used to influence the development of the infant’s immune system in such a way that allergies and autoimmune diseases later in life will be reduced or even eliminated.

## Conflict of Interest Statement

The authors declare that the research was conducted in the absence of any commercial or financial relationships that could be construed as a potential conflict of interest.
